# Direct Laser Writing of Selectively Degradable Polypeptide Hydrogel Microstructures by Proteolytic Enzymes

**DOI:** 10.1021/acsami.5c02960

**Published:** 2025-06-10

**Authors:** Yekaterina Tskhe, Viviane Chiaradia, Brian J. Rodriguez, Larisa Florea, Colm Delaney, Robert D. Murphy

**Affiliations:** † School of Chemistry, Trinity College Dublin, Dublin 2, Ireland; ‡ The SFI Centre for Advanced Materials and BioEngineering Research (AMBER), Trinity College Dublin, Dublin 2, Ireland; § Department of Chemistry, RCSI University of Medicine and Health Sciences, 123 St. Stephen’s Green, Dublin D02 YN77, Ireland; ∥ School of Physics and Conway Institute of Biomolecular and Biomedical Research, 8797 University College Dublin, Dublin 4 N2E5, Ireland

**Keywords:** polypeptides, hydrogels, direct laser writing, two-photon polymerization, enzyme degradation

## Abstract

The discovery of materials with on-demand, tunable degradability is of significant utility for (micro)­structures generated through additive manufacturing techniques. Disclosed here are a series of star polypeptide cross-linkers comprising either l-lysine alone or l-lysine and l-alanine residues for use in direct laser writing of hydrogel microstructures, which are selectively degradable via intrinsic amino acid affinities to different proteolytic enzymes. Through multimaterial printing, direct laser writing permitted the formation of microstructural topographies through free radical polymerization of formulated polypeptide cross-linkers and commercial photoresists, whereby sections of the microstructures could undergo selective degradation in the presence of target proteases, thermolysin, and trypsin.

## Graphical abstract

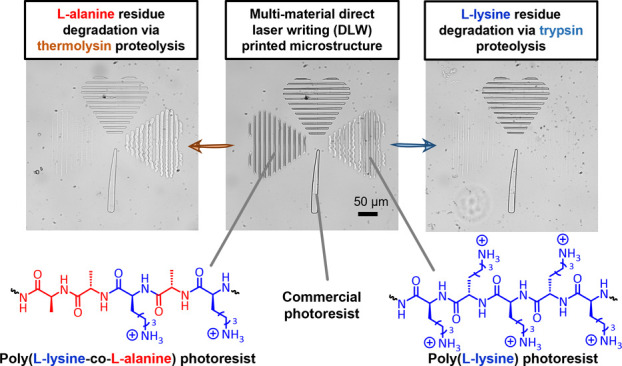

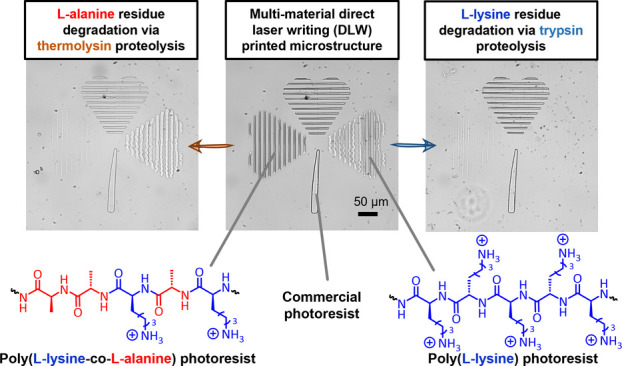

## 1 Introduction

Over the past decade, additive manufacturing (AM) has emerged as a vital fabrication tool for a range of fields from construction^1^ to biomedicine.^2^ AM has provided facile access to more complex geometries when compared to traditional methods including lithography or molding.^3^ Technological advancements toward light-based technologies such as digital light processing (DLP) and continuous liquid interface production (CLIP) have enabled users to generate highly complex 3D structures. To achieve microscale polymeric 3D structures with high resolution, particular focus has been given to direct laser writing (DLW), often termed two-photon lithography or two-photon polymerization (2PP). The technique proceeds whereby a near-IR femtosecond laser enables near-simultaneous two-photon absorption within a photocurable monomer mixture for the fabrication of microstructures with submicron resolution.^4^ Traditionally, DLW photoresists have most often comprised highly reactive (meth)­acryloyl-based monomers, due to their rapid photopolymerization kinetics and amenable mechanical properties. DLW can also be adapted for other chemistries, such as thiol–ene reactions,^5^ in addition to the inclusion of stimuli-responsive monomers within photoresists, which induce 3D structural transformations including shape morphing,^6^ bioinspired actuation,^7^ or degradation of specific sections.^8^ The field of 4D printing has grown significantly to include tunable and highly controlled microfabrication that has developed from static microstructures to now include mobile microrobots with helical, spiral, and propeller designs.^9^ The use of stimuli-responsive materials in DLW has also given rise to the fabrication of soft microrobots,^10^ controlled by various stimuli including light,^11–13^ temperature,^14–17^ pH,^18,19^ and magnetic/electric fields.^20^ Several examples utilizing DLW have also been presented for application in biomedical devices, where 3D scaffolds are employed for cell culture^21^ and tissue engineering.^22,23^ Complex 3D architectures achieved via DLW have been shown to enhance the mechanical properties and stability of such scaffolds to promote the cell growth initiated within the structures.^24^

Recently, additional functionality has also been brought to DLW substrates, giving the ability to selectively ‘erase’ sections or whole 3D microstructures using redox chemistry,^29^ light,^30^ temperature,^31^ and enzymatic degradation.^28,32^ Such responsiveness can offer users a desirable temporal handle which can be used in several ways, such as facilitating the removal of supports for complex 3D structures^33,34^ or providing a trigger to release therapeutic payloads from microneedle arrays.^35^ Newly emerging photoresists can capitalize on this programmed degradability through the use of labile chemical bonds.^36^ There has been less attention toward developing 3D microstructures with modular degradability, with few notable exceptions.^37,38^

Of the stimuli available, enzymatic degradation represents a highly coveted stimulus, where the protease degradability of endogenous amino acids may be exploited through polymer incorporation.^39^ Bioderived polypeptides such as gelatin (chemically modified as gelatin methacryloyl (GelMA)), or bovine serum albumin (BSA)^40,41^ have been previously used within DLW (Figure 1), whereby endogenous enzymes such as matrix metalloproteinase-2 (MMP-2) could degrade DLW structures of GelMA.^25,26^ Despite their benefits (biocompatibility), these polypeptides and proteins suffer from batch variability, having undefined chemical structures and polymer chain lengths. Moreover, they have broad protease specificity for degradation due to the presence of numerous amino acid residues and clusters within their structures. While efforts to narrow protease specificity have come in the form of peptide^27^ or amino acid-based cross-linkers,^28^ they require expensive, laborious synthesis, are water-insoluble and have limited mechanical properties. To circumvent these issues, synthetic polymer chemistry provides a route to create tailor-made polypeptides (or polyamino acids) through the *N*-carboxyanhydride ring-opening polymerization (NCA ROP). Polypeptides can be obtained with high scalability, batch reproducibility, and controllable molecular weights.^42^ Continued efforts in the field have improved the synthetic access and reaction speeds for both monomers and polymers,^43,44^ while a plethora of orthogonal chemistries can be conducted on the vast range of reactive side chains.^45^ These versatile properties have enabled their use as 3D printable feedstocks,^46^ while recently our group have reported how tailoring polymer topological design can facilitate DLW.^47^ Of distinct significance for new photoresist design, polypeptide degradation profiles can be readily tailored by judicious choice of amino acid. Embedding specific amino acid residues along a polymer backbone can therefore permit selective enzyme targeting of the designed polypeptide system.^48,49^ This concept is utilized to great effect herein, where the development and application of polypeptides 3D fabrication of microstructures via DLW is explored (Figure 1). Through a pragmatic design approach, homo- and copolypeptide cross-linkers, with selective affinities for two different enzymes, trypsin and thermolysin, were prepared as photoresists to generate microstructures and microtopologies with programmed degradability.

**Figure 1:**
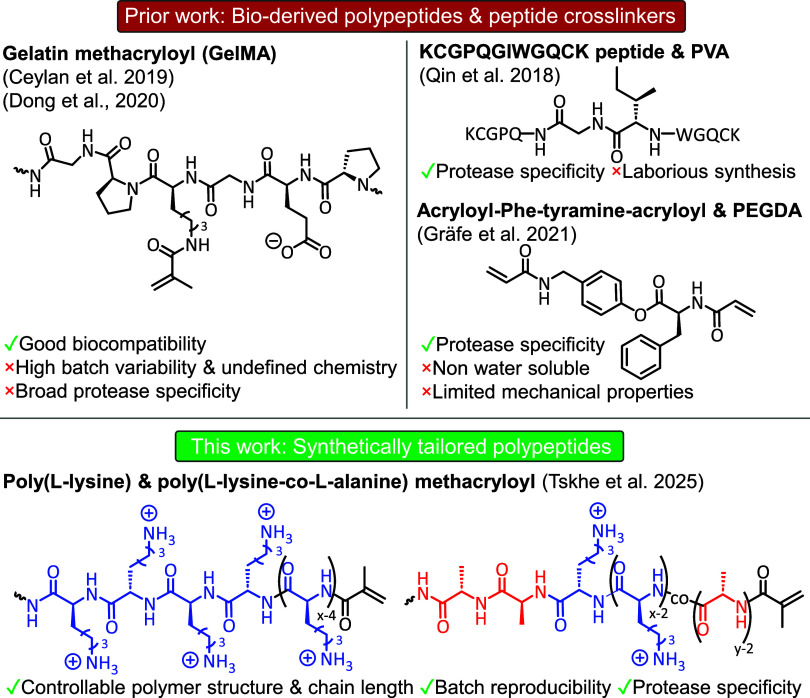
Scheme showing prior work on direct laser writing of enzyme-degradable peptide/polypeptides, and this work on modular synthetic polypeptides.^25–28^

## 2 Experimental Section

### 2.1 Materials

All chemicals were obtained from Sigma-Aldrich unless otherwise noted. Z-l-lysine, l-alanine, triphosgene, and 1,1,3,3-tetramethylguanidine (TMG) were purchased from Doug Discovery (Fluorochem). *N*-Hydroxyethylacrylamide (HEAAm) and the dialysis SnakeSkin membrane (3.5 kDa MWCO) were purchased from Thermo Fisher Scientific.

### 2.2 Methods

^1^H NMR spectra were recorded on a Bruker Avance 400 (400 MHz) spectrometer at room temperature by using TFA-d as a solvent. Attenuated total reflection (ATR) FTIR was recorded using a Thermo Scientific iS10 spectrometer in the region of 4000–600 cm^–1^. Initially, a background measurement was performed before analyzing the sample, with 16 scans completed using a resolution of 2 cm^–1^. Size exclusion chromatography (SEC) was performed in hexafluoroisopropanol (HFIP) using a PSS SECurity GPC system equipped with a PFG 7 μm 8 × 50 mm precolumn, a PSS 100 Å, 7 μm 8 × 300 mm, and a PSS 1000 Å, 7 μm 8 × 300 mm column in series and a differential refractive index (RI) detector at a flow rate of 1.0 mL min^–1^. The system was calibrated against Agilent Easi-Vial linear poly­(methyl methacrylate) (PMMA) standards and analyzed by the software package PSS winGPC UniChrom. Raman spectra for the examined microstructures were recorded using WITec alpha300 R Raman microscopy system (WITec, Oxford Instruments, Germany) with a 532 nm laser and 20x objective (NA 0.4). Integration times of 5 s and 20 accumulations were used for all measurements. No sample modification was performed prior to spectral acquisition. All SEM micrographs were captured using a Zeiss ULTRA plus microscope (Zeiss, Germany). For tilted SEM images, the samples were mounted on a 45 ° SEM mount pins (Ted Pella, Inc.). Prior to imaging, the samples were coated with Au/Pd layer of ∼15 nm using a sputter coater (Cressington Scientific Instruments, Watford, UK). All images were acquired under high vacuum using accelerating voltages of 5 kV.

### 2.3 4-Arm Star Poly­(Z-l-lysine) (4-PZLL_80_)

ZLL NCA (2.00 g, 6.53 mmol) was dissolved in 50 mL of CHCl_3_:DMF (5:1) in a round-bottom flask with stirring at room temperature. A G1 PPI dendrimer (34.50 mg, 10.89 × 10^–2^ mmol) in 1 mL of CHCl_3_ was then rapidly added to the solution. The reaction was continued until full consumption of NCA was confirmed by FTIR spectroscopy , followed by precipitation into 300 mL of diethyl ether and drying under vacuum (yield: 1.54 g, 88%). 4-Arm star poly­(Z-l-lysine-*co*-l-alanine) (4-PZLL_60_-*co*-LA_20_) was obtained using the same protocol (yield: 1.30 g, 81%).

### 2.4 4-Arm Star Poly­(l-lysine)-methacryloyl (4-PLL_80_MA – 4-P1)

The star polypeptide (1.50 g, 7.04 × 10^–2^ mmol) was dissolved in 60 mL of CHCl_3_:MeCN (5:1), followed by the addition of 1,1,3,3-tetramethylguanidine (TMG) (325 mg, 2.19 mmol) with stirring in an ice bath. Methacrylic anhydride (868.50 mg, 5.63 mmol) was then added dropwise, and the reaction was continued for 36 h in the dark, warming to room temperature. The polymer solution was then precipitated into diethyl ether and dried in vacuum. The functionalized star polypeptide was then dissolved in 20 mL of trifluoroacetic acid, and 5 mL of HBr (33 wt % in acetic acid) was added, and stirred for 18 h in the dark. The deprotected polymer was then precipitated into 200 mL of diethyl ether, redissolved in 15 mL of MeOH, and reprecipitated into diethyl ether twice. The polypeptide was then dried under vacuum to yield 4-P1 (yield: 0.935 g, 68%). 4-Arm star poly­(l-lysine-*co*-l-alanine)-methacryloyl (4-PLL_60_-*co*-LA_20_MA – 4-P2) was prepared in the same manner (yield: 0.980 g, 71%).

### 2.5 Rheology

Rheological measurements of photoresists were completed on an MCR 301 digital rheometer (Anton Paar). All experiments were conducted at room temperature (20 °C) using a parallel plate (PP25, Anton Paar) consisting of a 25 mm diameter geometry and a gap length of 0.095 mm. A protective hood was used to prevent water evaporation. For photorheology, a 405 nm visible light LED (M405L3-C1, Thorlabs) was installed to conduct photo-cross-linking experiments (intensity 6 mW cm^–2^). Formulations were prepared as described below (Section 2.6).

### 2.6 Photoresist Resin Preparation

The cross-linkers (4-P1 and 4-P2) were mixed with HEAAm comonomer and LAP photoinitiator, respectively, in order to prepare the photoresist for DLW. The preparation steps for both 4-P1 and 4-P2 photoresists were optimized to fully dissolve the components. The 4-P1 (Table S3) homopolypeptide (126.1 mg) was added to HEAAm (95.5 mg) and deionized water (102 μL), and mixed at room temperature. Subsequently, the mixture was heated using a heat gun and placed into a centrifuge for 10 min. Lastly, LAP (12.5 mg) was added, followed by further mixing. The same procedure was repeated for 4-P2, following the formulation described in Table S4.

### 2.7 Direct Laser Writing (DLW)

DLW fabrication was performed using a Nanoscribe GT Photonic Professional system (Nanoscribe GmbH) with a 50 mW femtosecond laser at 780 nm. All experiments were carried out in an oil immersion configuration using a 63X objective (Zeiss, Plan Apochromat) and a drop of oil (Zeiss Immersol 518F). The glass slide substrates (30 mm diameter, 0.16 to 0.19 mm thickness) (Thermo Fisher Scientific) were silanized prior to DLW fabrication process to achieve covalent attachment of the microstructures. A three-step fabrication process was established to obtain multimaterial microstructures composed of nondegradable commercial IP-L780 photoresist (Nanoscribe GmbH) and enzyme-degradable parts composed of 4-P1 and 4-P2 photoresists. As the first step, the nondegradable cross-shaped markers were fabricated for further alignment of polypeptide-based components (Figure S15). As displayed in Figures 5 and 6, nondegradable parts were also part of the design in order to confirm the degradation of components fabricated with polypeptide-based photoresists, while nondegradable parts would remain untouched. The IP-L780 microstructures were immersed in propylene glycol monomethyl ether acetate (PGMEA) for 15 min, followed by immersion in isopropanol (IPA) for 3 min. For the next step, two PSA strips were placed on the slide with a smaller top coverslip (12 mm diameter, 0.170 ± 0.005 mm thickness). One drop of 4-P1 photoresist was placed near the center of the coverslip. For the development of 4-P1-based microstructures, IPA:H_2_O (70:30 (v/v)) at 60 °C was used in order to remove nonpolymerized parts of photoresist. The same procedure was followed for the fabrication of 4-P2 microstructures. Lastly, the sample was dried under N_2_ and placed into a well plate to prevent contamination.

### 2.8 Degradation of DLW Printed Structures

Degradation of DLW samples containing 3 materials, namely, IP-L780, 4-P1, and 4-P2, was performed using optical microscopy imaging (Olympus IX81 motorized inverted microscope). All images were collected in bright-field mode. The selective degradation of both polypeptides was observed in thermolysin solution (50 mM tris (pH 8.0), 0.5 mM CaCl_2_, 0.25 mg/mL enzyme) and trypsin solution (50 mM tris (pH 8.0), 0.25 mg/mL enzyme and 1 mg/mL). Time-resolved studies were performed in a thermolysin solution until full degradation was observed. The degradation of all fabricated microstructures was conducted under aqueous conditions. In the constructed cell with PSA strips used as spacers and a top coverslip (12 mm diameter, 0.170 ± 0.005 mm thickness), two drops of prepared enzyme solution were added. The degradation in trypsin solutions was performed overnight in a sealed well plate. The quantification of degradation over time was analyzed using ImageJ software by measuring the total area of microstructure at each time interval.

## 3 Results and Discussion

### 3.1 Polypeptide Preparation

*N*-Carboxyanhydride (NCA) monomers of carbobenzyloxy-l-lysine (ZLL) and l-alanine (LA) were synthesized using epichlorohydrin as an HCl scavenger.^50^ Monomers were made in high yield and of high purity, confirmed via ^1^H NMR and FTIR spectroscopy (Figures S1–S4). To prepare the polymers, NCA ZLL or a mixture of NCA ZLL/NCA LA underwent controlled ring-opening polymerization (ROP) in the presence of a generation one (G1) four-armed poly­(propyleneimine) (PPI) dendrimer to yield star homopolypeptides or copolypeptides (Figure 2A) with full conversion confirmed with FTIR and ^1^H NMR spectroscopy. The total degree of polymerization (DP) was maintained at 80 for both systems, forming a star poly­(Z-l-lysine) (4-P­(ZLL_80_)) and star poly­(Z-l-lysine-*co*-l-alanine) (4-P­(ZLL_60_-*co*-LA_20_)), with a total of 20 units per arm, respectively. Experimental DPs and polymer compositions were confirmed by ^1^H and DOSY NMR spectroscopy (Figures S5–S7), with DPs of 80 for the homopolymer and 60/20 for the copolymer, while size exclusion chromatography (SEC) confirmed molecular weights (*M*_n_) and low dispersities (*Đ*) (Figure S8, Table S1). Polymer chain ends were then functionalized with methacryloyl groups, using methacrylic anhydride and tetramethylguanidine (TMG). The vinyl and methyl groups from methacryloyl moieties were observable at 5.75–6.00 and 1.76 ppm on ^1^H NMR spectra (Figures S9 and S10). The Z groups were then removed through acid hydrolysis, leaving free lysine amino residues as confirmed by ^1^H NMR spectroscopy (Figures S11 and S12). Formed poly­(l-lysine) (4-P­(LL_80_)­MA) and poly­(l-lysine-*co*-l-alanine) (4-P­(LL_60_-*co*-LA_20_)­MA) cross-linkers were referred to as 4-P1 and 4-P2 herein. For enzyme responsiveness, it was anticipated that both polypeptide cross-linkers would degrade with trypsin due to the presence of l-lysine,^51^ while the inserted l-alanine residues on the copolypeptide would facilitate degradation using thermolysin.^52^ Determination of any distinguishable secondary structures for the polypeptides was confirmed via FTIR spectroscopy,^53^ to examine the influence of alanine residues (Figure S13). Within amide I, both the homopolypeptide and copolypeptide exhibited multiple absorbance bands, while identical amide II bands were noted. Absorbances at 1651 cm^–1^ are anticipated to be α-helical structures from unordered lysine sequences, observable for both 4-P1 and 4-P2. Shoulder features noted for both at 1665 and 1637 cm^–1^ are characteristic of mixed random and sheet-like conformations,^53^ which confirms that l-alanine residues do not appear to influence secondary structures.

### 3.2 Rheology and Direct Laser Writing

Each star polypeptide (4-P1, 4-P2) was formulated into an aqueous resin with an *N*-hydroxyethylacrylamide (HEAAm) comonomer and a lithium phenyl-2,4,6-trimethylbenzoylphosphinate (LAP) photoinitiator (Figure 2A). HEAAm was chosen as it facilitates rapid photopolymerization kinetics, endowing good structural resolution to DLW printed objects, as disclosed in our previous study.^47^ Photo-cross-linking regimes were then tracked in real time using 405 nm visible light irradiation (Figure 2B). After a 90 s incubation time, photo-cross-linking was initiated using a collimated light source (405 nm, 6 mW/cm^2^), with both resins evolving from initially fluid-like properties to solid viscoelastic properties. The characteristic gel point crossover of the storage (*G*′) and loss modulus (G″) was observed within 10–15 s for both 4-PL1 (Figure 2C) and 4-PL2 (Figure 2D). The *G*′ modulus begins to reach a plateau at 220 s for 4-P1, while interestingly for 4-P2, an incremental increase in *G*′ is still observed after this time. Mechanically robust hydrogels are formed, having *G*′ of 5360 and 7520 Pa for 4-P1 and 4-P2, respectively. The incorporation of alanine does appear to slightly influence hydrogel mechanical properties, evidenced by ∼2140 Pa difference in storage modulus values, which may be due to increased hydrophobicity. More comparative results were observed from a rheological stress-strain sweep, with similar yield stress values (95.7 and 97.6 Pa), and yield strain values (2326% and 2720%) for 4-P1 (Figure S14A) and 4-P2 (Figure S14B), respectively. Similar equilibrium swelling ratios (Q) were also observed for 4-P1 (19.7) and 4-P2 (21.7) cross-linked networks (Table S2).

**Figure 2:**
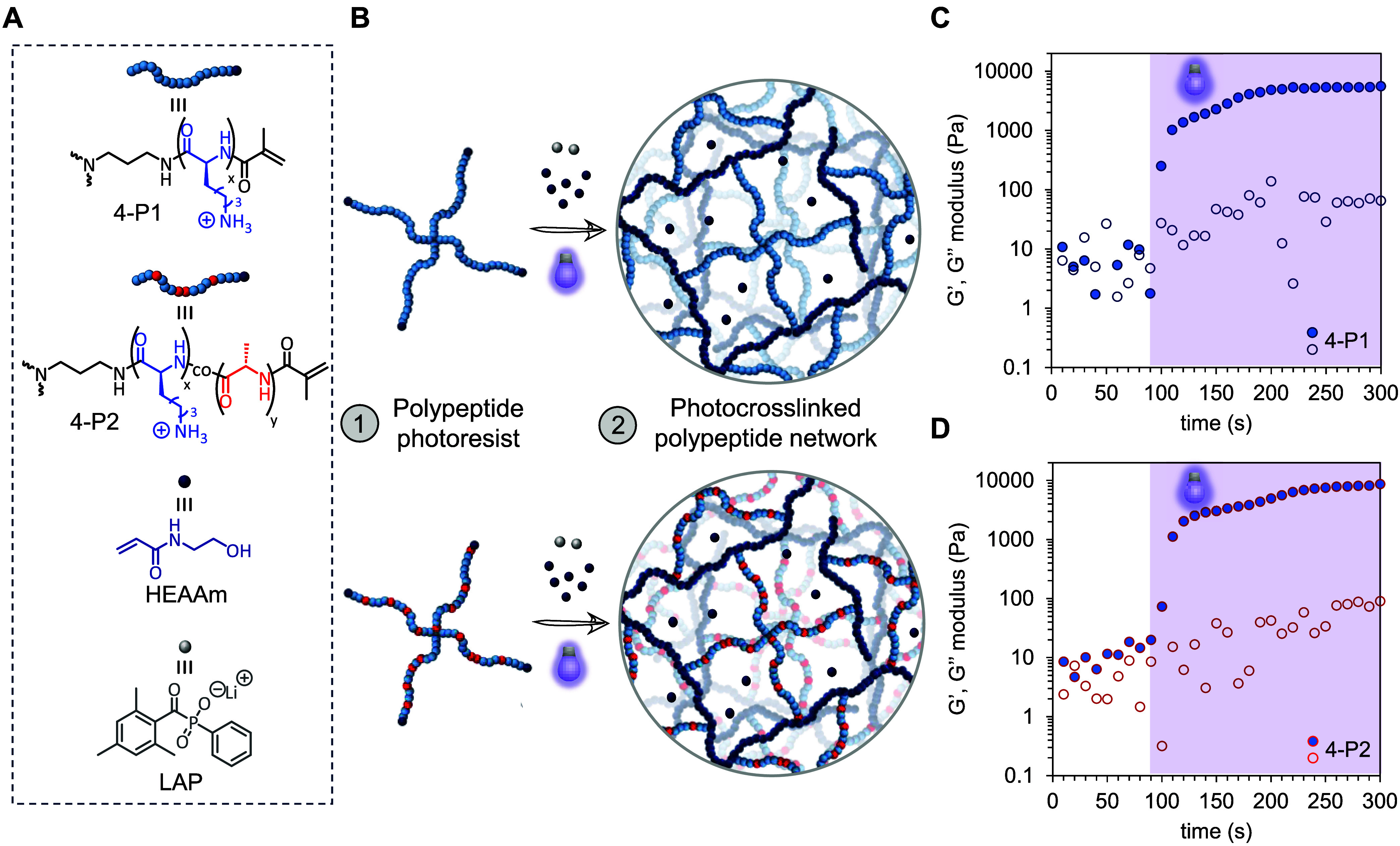
Graphical representation of polypeptide photoresist (A) comprising star polypeptide cross-linkers (4-P1 and 4-P2), LAP photoinitiator, and HEAAm comonomer; and (B) visible light photopolymerization forming photo-cross-linked polypeptide networks. (C,D) Rheological time sweep displaying photopolymerization kinetics of the 4-P1 and 4-P2.

DLW enabled 3D microfabrication from polypeptide photoresists. In this process, multiple photon absorption can occur via a so-called ‘virtual’ state, which exists for a few femtoseconds. Outside the focal point of the femtosecond laser, the photoresist and photoinitiator remain unperturbed. Within the confines of this small volume, known as a voxel (Figure 3A), photoinitiators undergo excitation, from ground state (S_0_) to an excited singlet state (S_1_) via the virtual state (Figure 3B). The typical path involves intersystem crossing (ISC), then radiative relaxation, phosphoresce, or homolytic cleavage with the formation of a radical initiator, initiating the polymerization process.^4^ Exploiting this strategy, both 4-P1 and 4-P2 cross-linkers were formulated with comonomer and photoinitiator, to prepare photoresists of the same name (Tables S3 and S4), guided by resin formulations in our previous DLW study.^47^ Microscale lines (Figure 3D,G) and roses (Figure 3F,I) were fabricated using 4-P1 and 4-P2 and imaged by scanning electron microscopy (SEM). Optimization of the fabrication process was achieved by adjusting parameters such as the laser power, scanning speed, slicing, and hatching (Figure S15). Relatively low laser powers were required to print 4-P2 photoresists (35.0 mW), while slightly higher laser powers were required to print resolved 4-P1 microstructures (42.5 mW), though a wide variety of structures were achievable for both photoresists. A series of microscale grids fabricated with both photoresists revealed the similar printing quality achieved when varying laser power (20–35 mW) and scan speeds (8000, 9000, 10,000 μm s^–1^) (Figures S16–S18). Other microstructures were also demonstrated using 4P-1 or 4P-2 photoresists to achieve 3D structures containing overhangs or suspended parts (Figure S19). Chemical composition of fabricated hydrogel microstructures was confirmed by Raman spectroscopy (Figure 3E,H) with peaks observed relating to amide I (1649, 1655 cm^–1^) and amide II (1452, 1457 cm^–1^) bands for 4-P1 and 4-P2, respectively, while full assignment of peaks is available in Figure S20.

**Figure 3:**
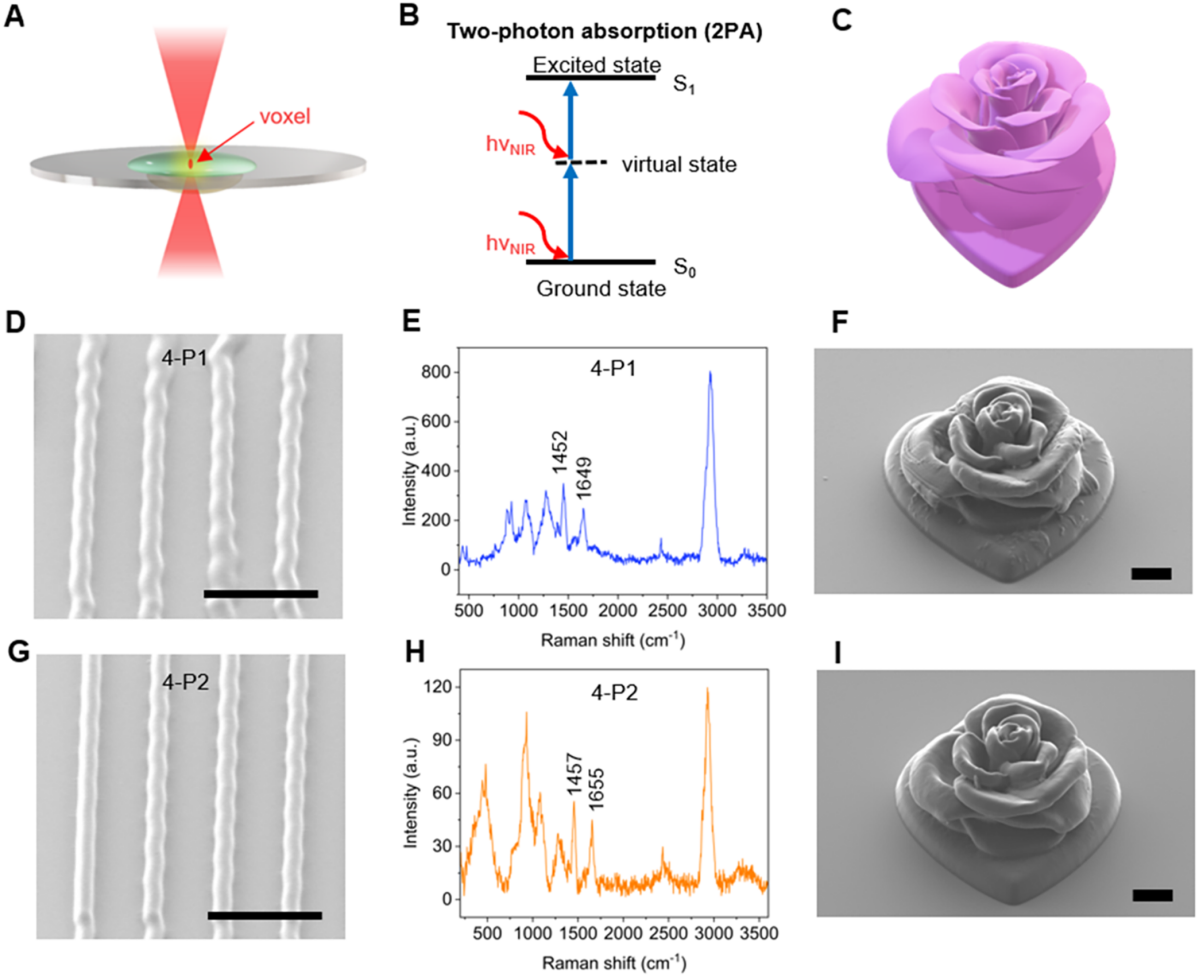
(A) Schematic of DLW within photoresist droplet. (B) Simplified Jablonski diagram for the principle of two-photon absorption. (C) 3D CAD design of microscale rose. (D, G) SEM images of DLW-fabricated microscale lines composed of 4-P1 and 4-P2 hydrogel networks, respectively (scale bar: 20 μm). (E, H) Raman spectra of the corresponding microstructures of 4-P1 and 4-P2. (F, I) SEM images of DLW-fabricated microscale rose composed of 4-P1 and 4-P2 hydrogel networks, respectively (scale bar: 10 μm).

Characterization of the resulting microstructures was performed using atomic force microscopy (AFM). First, this was used to provide information on the effect of applied laser power on the height of the resulting microstructures. An array of cubes with design dimensions 10 × 10 × 4 μm were fabricated with laser powers ranging from 20 to 40 mW, at a constant scan speed of 10,000 μm s^–1^ (Figure 4A–C,E,F). As fabricated, the maximum height for 4P-1 cubes was 3.56 ± 0.08 and 3.13 ± 0.03 μm for 4P-2 cubes, both achieved with a laser power of 37.5 mW. When hydrated in deionized water, all cubes were seen to increase significantly in height. (Figure 4D).

**Figure 4:**
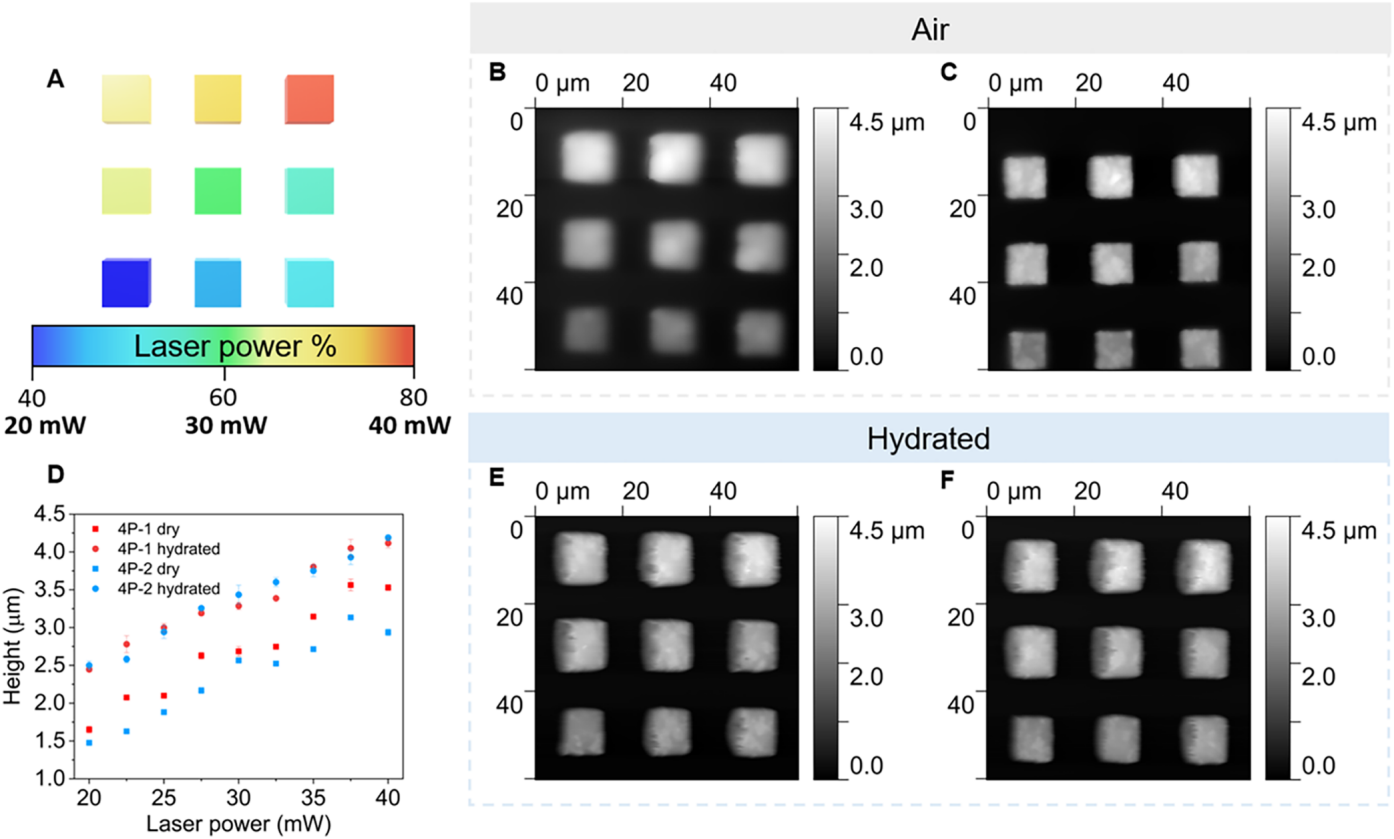
AFM analysis of cube array. (A) Array design highlighting the range of laser powers employed for fabrication. Height topography of (B) 4P-1 and (C) 4P-2 in air. (D) Plot of height dependence on laser power for dry and hydrated cubes composed of 4P-1 and 4P-2 photoresists. Height topography of hydrated (E) 4P-1 and (F) 4P-2 cube array.

The same cube arrays were utilized to probe their mechanical properties. Using AFM, Young’s modulus values were obtained from force curves measured for each cube fabricated at each laser power. For the applied range of laser powers (20–40 mW), the Young’s modulus values in air were in GPa range for both materials. For example, at 32.5 mW laser power, a Young’s modulus of 4.13 ± 0.35 and 2.48 ± 0.10 GPa was measured for 4P-1 and 4P-2 cubes, respectively. Upon hydration, a decrease of Young’s modulus by 2 orders of magnitude to MPa range was observed for the 4P-1 cube array. For example, a cube fabricated at 27.5 mW laser power showed a Young’s modulus of 2.09 ± 0.32 GPa in air, and this decreased to 32.59 ± 3.99 MPa upon hydration.

### 3.3 Microstructure Degradation

Degradation characteristics of the 4-P1 and 4-P2 microstructures were then determined via enzyme proteolysis. Trypsin was chosen as a suitable enzyme for both polypeptides, as it is known to cleave adjacent to basic amino acid residues such as lysine,^54^ a residue present in both the homopolypeptide (4-P1) and copolypeptide (4-P2) cross-linkers. Alternatively, thermolysin has the ability to target alanine residues,^55^ exclusively part of the copolypeptide (4-P2). Studies were completed on DLW printed lines of the following design: 150 μm in length, 2.5 μm in width, and 3 μm in height. A commercial acrylate-based photoresist, IP-L780, was printed as a control, and the other two structures were fabricated from 4-P1 and 4-P2, respectively (Figure 5A), with microstructures confirmed by SEM (Figure 5B). Initially, degradation of 4-P2 patterns using thermolysin was conducted to illustrate how polypeptide design can permit selective degradability. DLW printed lines from 4-P2 were seen to instantaneously swell in water via optical microscopy. Real-time 4-P2 degradation was then tracked and quantified in over 5 min intervals, using ImageJ software to analyze size changes. The initial total area of 4-P2 lines was 4155 ± 61 μm^2^ in the dry state, swelling to 6467 ± 169 μm^2^ after addition of the thermolysin solution. After this point, a rapid decrease in the total area was measured. Degradation was observed after 150 min (Figure 5D,E), with a final total area of 566 ± 14 μm^2^ recorded. No visible microstructures remained on the slide surface, only outlines of programmed designs, which were further confirmed by SEM (Figure 5C).

**Figure 5:**
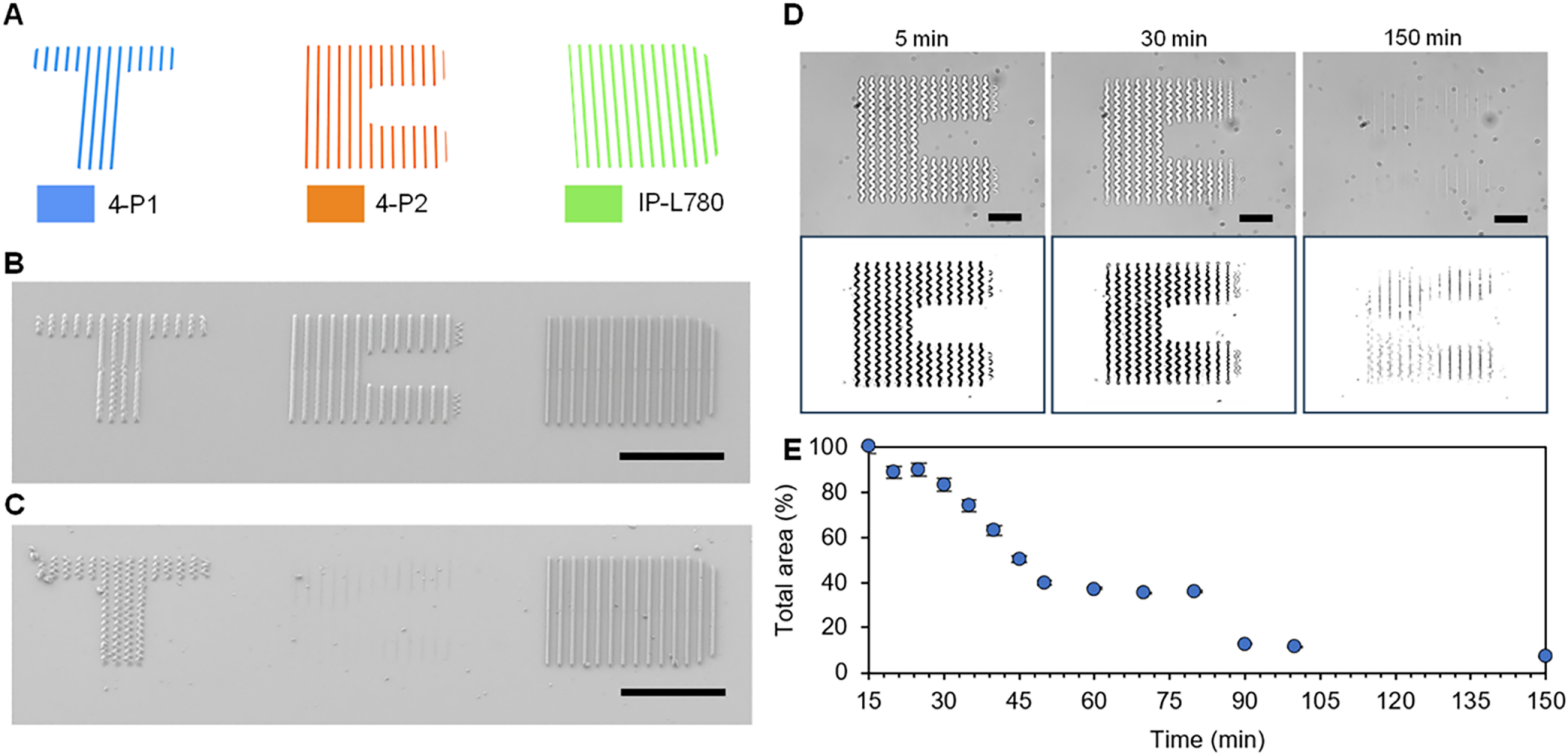
(A) 3D CAD design of TCD letters. SEM image of microstructure pre-degradation (B) and (C) post-degradation (scale bar: 100 μm). (D) Degradation of 4-P2 microstructures in thermolysin solution (0.25 mg/mL) over time, highlighting degraded area (scale bar: 50 μm). (E) Graph showing decrease of total area of microstructures (*n* = 3) (scale bar: 50 μm). Fabrication parameters: 4-P1 (35 mW, 10,000 μm s^–1^), 4-P2 (35 mW, 10,000 μm s^–1^), IP-L780 (40 mW, 10,000 μm s^–1^).

Multimaterial printing for sequential selective degradation of three-leaved clover microstructures (Figure 6A) was then demonstrated for 4-P2 (Figure 6B) and 4-P1 (Figure 6C) cross-linked hydrogel networks. Degradation of 4-P2 features from initial microstructures (Figure 6D) was again executed using thermolysin (Figure 6E), while 4-P1 structures remained untouched, highlighting the remarkable selectivity of the degradation process (Figure S22). Remarkably, less than 10% of the total area of 4-P2 remained after 150 min (Figure S23A–C), where real-time analysis showed the rapid degradation (Figure S24). Subsequent degradation of 4-P1 microstructures was then achieved in trypsin over a 24 h period (Figure 6F). To ensure full degradation after the incubation in enzyme solutions, samples were imaged using SEM, showing polypeptide microstructures before (Figure S25A) and after enzymatic degradation (Figure S25B,C). Degradation in thermolysin following the same time frame was also demonstrated for 3D encapsulation (Figure S26). In this case, a 4P-2 cube structure was degraded over 2.5 h to reveal an encased IP-L star (Figure S26). In comparison to other examples of enzymatically degradable microstructures made from GelMA or peptide cross-linkers, the polymer design disclosed here offers materials with controllable molecular weights, higher batch reproducibility, and far more protease-selective degradation regimes. The modular properties of these synthetic polypeptides render them ideal candidates for degradation-oriented applications in biomedical science, agriculture, and beyond.

**Figure 6:**
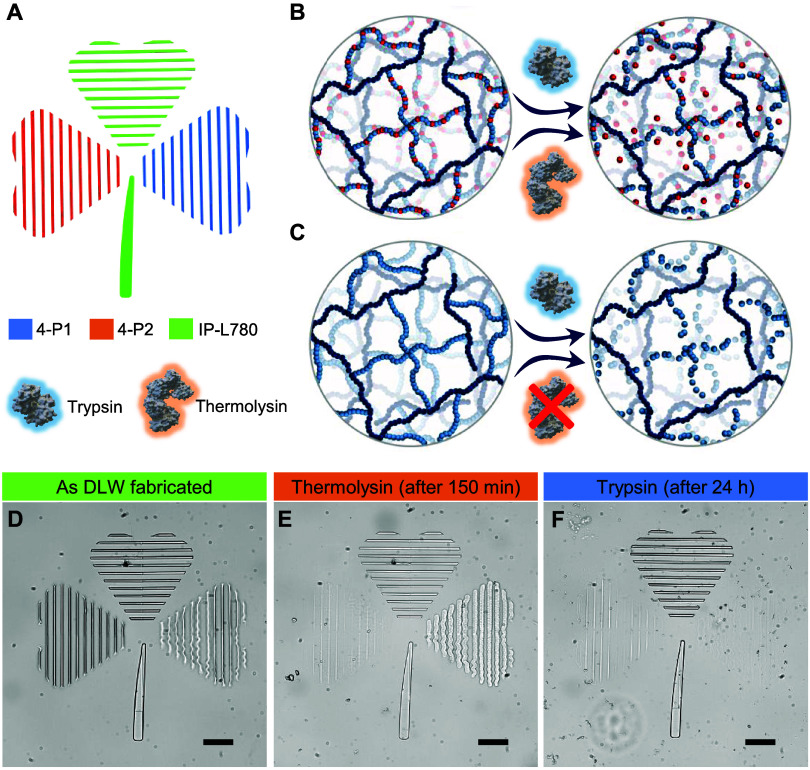
(A) 3D CAD design of a three-leaved clover. Schematic degradation of cross-linked 4-P2 hydrogel network in thermolysin or trypsin solution (B), and cross-linked 4-P1 hydrogel network in trypsin solution (C). Optical microscope images of fabricated microstructures (D), after incubation with thermolysin (E), and after incubation with trypsin (F) (scale bar: 50 μm). Fabrication parameters: 4-P1 (35 mW, 10,000 μm s^–1^), 4-P2 (35 mW, 10,000 μm s^–1^), IP-L780 (40 mW, 10,000 μm s^–1^).

## 4 Conclusions

A new series of degradable star polypeptide cross-linkers have been disclosed, which demonstrate selective proteolysis through the strategic incorporation of l-alanine residues. The cross-linkers were readily adapted for use in DLW 3D printing, showing the ability to generate arrays of features with micron-sized topographies. After characterizing selective proteolytic degradation of both materials separately, in thermolysin and trypsin, multimaterial fabrication was exploited to show the ability to form complex designs with selective degradation, over controllable time scales, juxtaposed with the commercial photoresist which remains unperturbed throughout the process. The high tunability of these materials is anticipated to serve as a canvas for bringing adaptable polypeptide chemistry to the realm of 4D fabrication.

## Supplementary Material



## Notes

Synthesis and characterization of NCAs, swelling ratio, AFM measurements for Young’s modulus, NMR and FTIR spectra, SEC plot, rheology plots, microscopy, and SEM images of DLW structures (PDF)
