# Supplementary material for: Direct Laser Writing of Selectively Degradable Polypeptide Hydrogel Microstructures by Proteolytic Enzymes
Source: ACS Appl Mater Interfaces. 2025 Jun 10;17(25):37043–52. doi: 10.1021/acsami.5c02960 (PMC12203468; doi:10.1021/acsami.5c02960)
Supplement: Supplementary file 1 [file am5c02960_si_001.pdf]

Supporting Information

Direct Laser Writing of Selectively Degradable Polypeptide Hydrogel Microstructures by Proteolytic Enzymes

Yekaterina Tskhe‡[1,2], Viviane Chiaradia‡[3], Brian J. Rodriguez,[4] Larisa Florea[1,2], Colm Delaney*[1,2], Robert D. Murphy*[3]

[1] School of Chemistry, Trinity College Dublin, Dublin 2, Ireland

[2] The SFI Centre for Advanced Materials and BioEngineering Research (AMBER), Trinity College Dublin, Dublin 2, Ireland

[3] Department of Chemistry, RCSI University of Medicine and Health Sciences, 123 St. Stephen's Green, Dublin 2, Ireland

[4] School of Physics and Conway Institute of Biomolecular and Biomedical Research, University College Dublin, Dublin, Ireland

‡**Equal contribution**: Yekaterina Tskhe, Viviane Chiaradia
***Corresponding authors**: Colm Delaney (cdelane5@tcd.ie), Robert D. Murphy (robertdmurphy@rcsi.com)

1. Additional methods

1.1. Z-L-lysine N-carboxyanhydride (ZLL NCA). Triphosgene (10.60 g, 3.57×10^1 mmol) and (±)-epichlorohydrin (26.40 g, 28.54×10^1 mmol) were dissolved in 200 mL THF in a round-bottomed flask with stirring. Z-L-lysine (20.00 g, 7.13×10^1 mmol) was then added and the reaction suspension was heated under reflux (70 °C). Reaction stirring and temperature were maintained until all solids disappeared and the solution became translucent (2-2.5 h). The solution was then cooled under an argon balloon, any unreacted Z-L-lysine was filtered off, and 2/3 of the THF solvent was removed by distillation. ZLL NCA was then precipitated by addition of 400 mL of hexane and stored overnight in a freezer (-18 °C) to maximise precipitation. The solid ZLL NCA was then filtered and dried under vacuum. It was then re-dissolved in 200 mL ethyl acetate, and precipitated into 350 mL of hexane, filtered and dried (this was completed two to three times). It was then dried extensively under vacuum to afford a white powder (yield: 15.20 g, 69%). ^{1}H NMR (400 MHz, $CDCl_3$/d-TFA, δ, ppm): 7.38 (m, 5H, -CH2C6*H5*), 5.17 (s, 2H, -C*H2*C5H6), 4.40 (t, 1H, -COC*H*NH-), 3.25 (m, 2H, -CH2C*H*2NH-), 1.90 (m, 2H, --CHC*H2*CH2-), 1.49 (m, 4H, -CHCH2*CH2CH*2-).

1.2. L-alanine N-carboxyanhydride (LA NCA). Triphosgene (7.57 g, 2.80×10^1 mmol) and (±)-epichlorohydrin (20.77 g, 22.45×10^1 mmol) were dissolved in 80 mL THF in a round-bottomed flask with stirring. L-alanine (5.00 g, 5.61×10^1 mmol) was then added and the reaction suspension was heated under reflux (70 °C). Reaction stirring and temperature were maintained until all solids disappeared and the solution became translucent (1-1.5 h). The solution was then cooled under an argon balloon and 2/3 of THF was removed by distillation. Crude LA NCA was then precipitated by addition of 250 mL of hexane, followed by storage overnight in a freezer (-18 °C). The solid was filtered, dried under vacuum, and re-dissolved in 80 mL ethyl acetate, and precipitated into 250 mL of hexane, followed by storage overnight in a freezer (-18 °C). The powder was filtered and dried extensively under vacuum to afford a cotton like white solid (yield: 4.10 g, 64%). ^{1}H NMR (400 MHz, DMSO-d, δ, ppm): 9.00 (s, 1H, N*H*), 4.46 (q, 1H, -COC*H*NH-), 1.3 (d, 3H, -CHC*H3*).

1.3. Swelling ratio. The synthesised star polypeptide (4-P1) and copolypeptide (4-P2) crosslinkers (10 wt%) were mixed with comonomer (10 wt%) and photoinitiator (0.1 wt%), *N*-(2-hydroxyethyl)acrylamide (HEAAm) and lithium phenyl-2,4,6-trimethylbenzoylphosphinate (LAP), respectively, in order to prepare the hydrogel formulation. Photocrosslinking was conducted using a 405 nm visible light LED (M405L3-C1, Thorlabs), irradiating the formulations for 10 mins (intensity 6 mW cm^{-2}). Photocrosslinked hydrogels were then washed with DI water and lyophilised. After the dry mass was recorded, the hydrogels were then swollen in excess water for 24 h to ensure full equilibration of the networks. The hydrogel was then removed from the water, patted dry to remove

excess water on the surface and the mass was recorded again. The procedure was conducted in triplicate. The equilibrium swelling ratio (Q) was calculated based on equation S1:

$$\boldsymbol{Swelling\ Ratio} = \frac{\boldsymbol{Ws-Wd}}{\boldsymbol{Wd}}$$

Where ***Ws****=mass of swollen hydrogel and* ***Wd****=mass of pre-swollen hydrogel.*

1.4. Atomic force microscopy (AFM). The measurements were obtained using MFP-3D (Asylum research) system. The imaging in air and deionised water was perfomed in AC (tapping) mode. The PPP-EFM probe (Nanosensors) with tip radius 25 nm, resonance peak around 75 kHz and spring constant of 2.8 N m^{-1} was used for imaging in air. The Electrical all-in-one probe (BudgetSensors) with tip radius 25 nm, resonance peak around 80 kHz and spring constant of 2.7 N m^{-1} was used for imaging in deionised water. The array of cubes with the dimensions of 10 x 10 x 4 µm was imaged with 256 lines per scan and scan size of 60 x 60 µm. Scan angle of 0° and scan rate of 0.3 Hz was used. Height topography images were further processed using Gwyddion software (version 2.62). The reported height value of a single cube fabricated with a specific laser power was an average of 5 height values. The Young's modulus values were obtained from force curves measured for cube fabricated with laser powers of 20 – 40 mW on contact mode. The Tap300-G probe (BudgetSensors) with tip radius 10 nm, resonance peak around 300 kHz and spring constant of 40 N m^{-1} was used for all force curve measurements. Probe was calibrated for deflection InvOLS (inverse optical lever sensitivity) on a glass slide. The fitting of the extension segments of force curve was done for Hertz model using Asylum software. The model assumes a conical tip geometry with a half-angle of 20° and the sample Poisson's ratio of 0.45. Each Young's modulus value is an average of three continuous force curves measured for all cubes in the array.

2. Additional figures

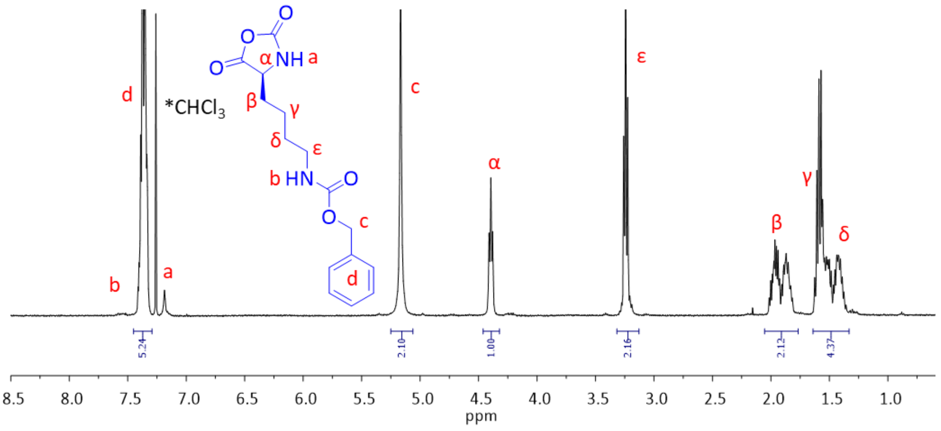

Figure S1. ^{1}H NMR spectrum of ZLL NCA (400 MHz, $CDCl_3$/TFA-d).

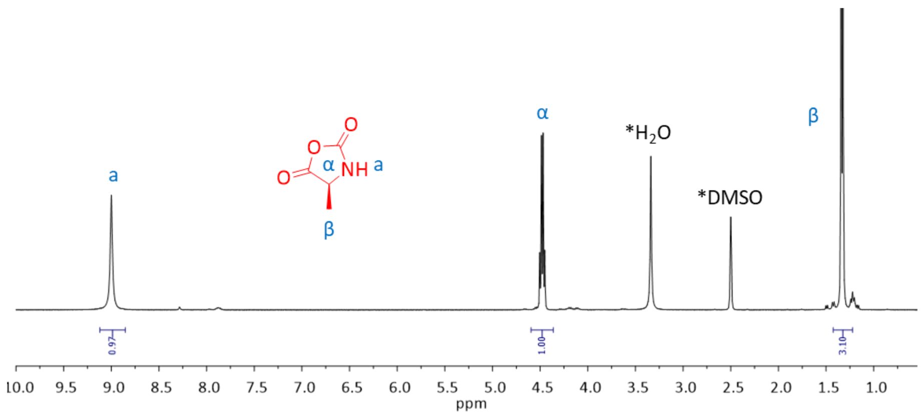

Figure S2. ^{1}H NMR spectrum of LA NCA (400 MHz, DMSO-d).

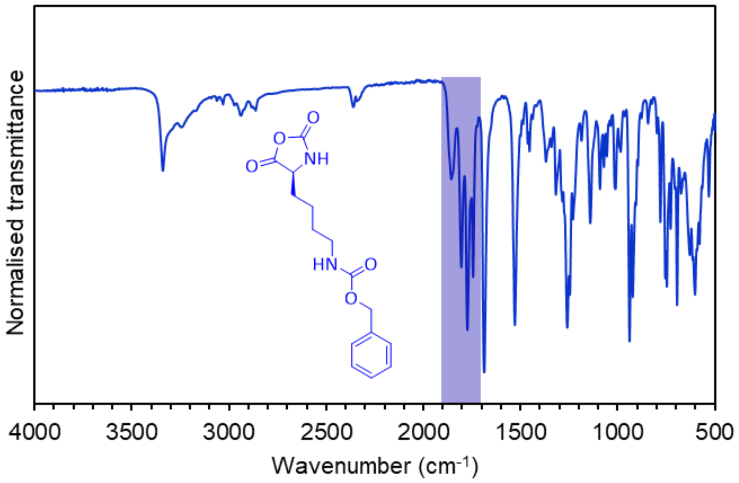

Figure S3. FTIR spectrum of ZLL NCA (highlighting anhydride rings).

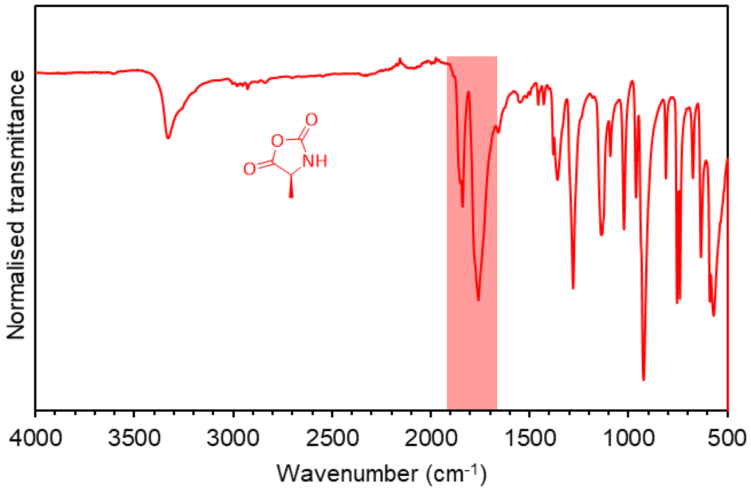

Figure S4. FTIR spectrum of LA NCA (highlighting anhydride rings).

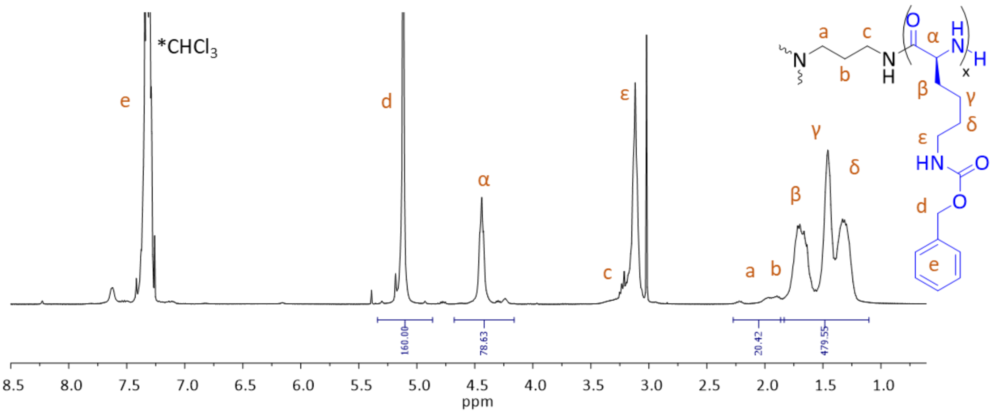

Figure S5. ^{1}H NMR spectrum of 4-P(ZLL)$_{80}$ (400 MHz, $CDCl_3$/TFA-d).

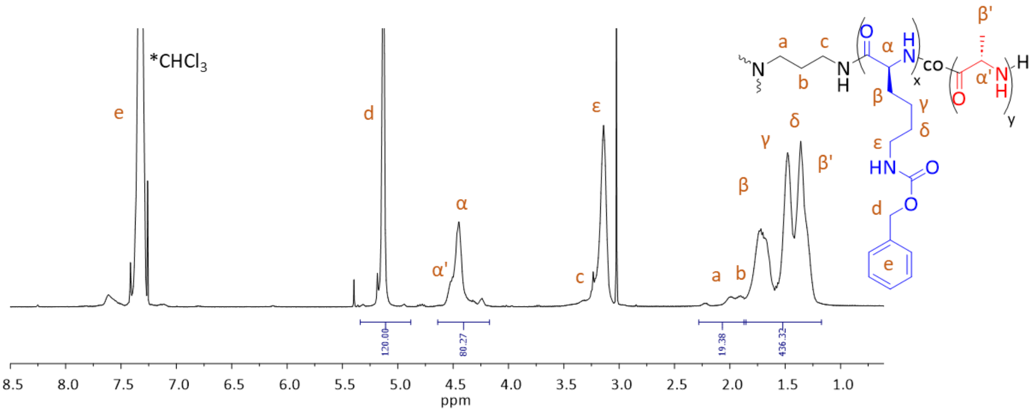

Figure S6. ^{1}H NMR spectrum of 4-P(ZLL$_{60}$-*co*-LA$_{20}$) (400 MHz,$CDCl_3$/TFA-d).

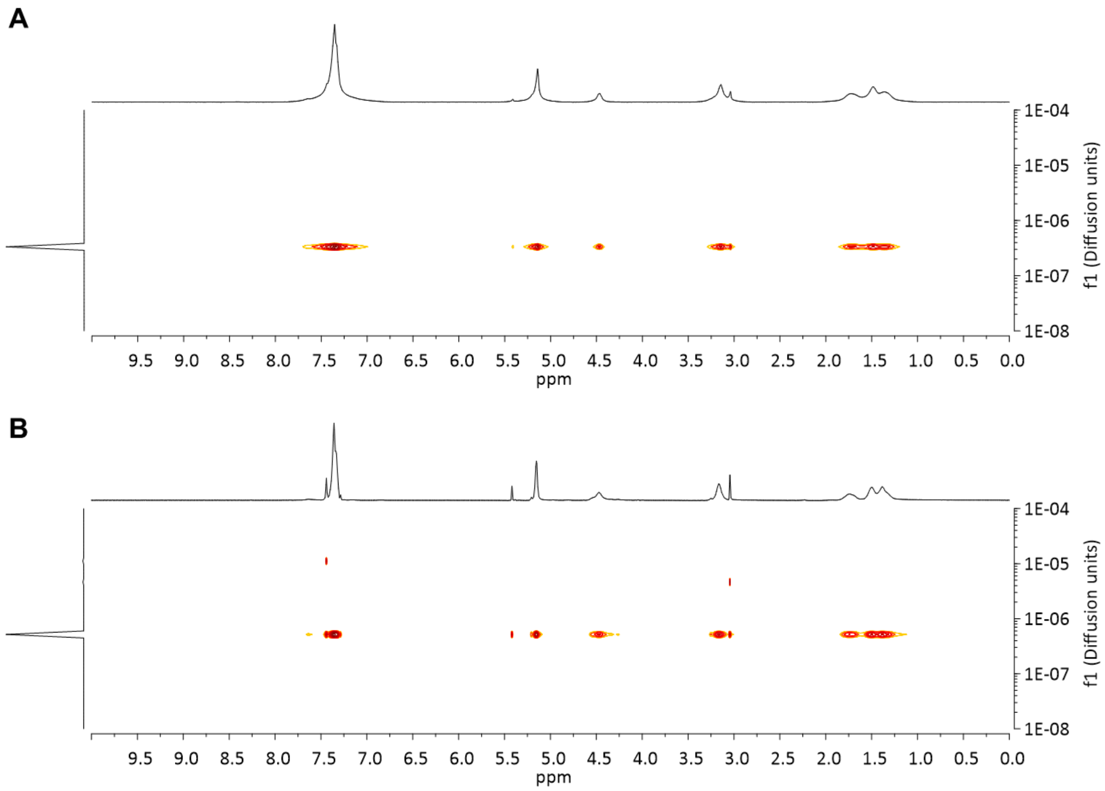

Figure S7. DOSY NMR spectra of 4-P(ZLL$_{80}$) (A) and 4-P(ZLL$_{60}$-*co*-LA$_{20}$) (B) (400 MHz, $CDCl_3$/TFA-d).

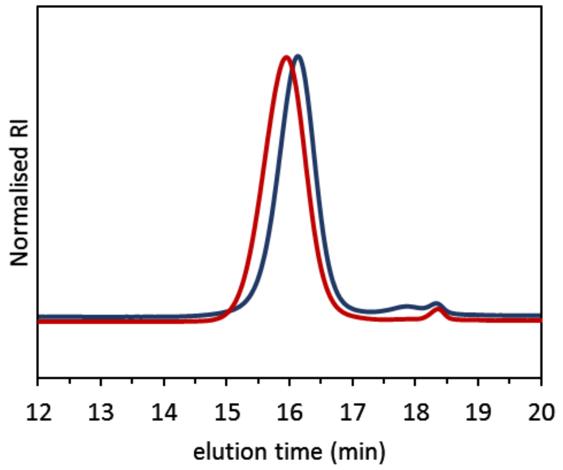

Figure S8. SEC traces of 4-P(ZLL$_{80}$) (red) and 4-P(ZLL$_{60}$-*co*-LA$_{20}$) (blue).

Table S1. Degree of polymerization (DP) and molecular weight characteristics of 4-arm homopolypeptides and copolypeptides.

Polymer	DP[a]	DP[b]	M_n[a] (g/mol)	M_n[b] (g/mol)	M_n[c] (g/mol)	$Đ_M$[c]	Diffusion coefficient[d]
4-P(ZLL_{80})	80	80	21300	21300	37900	1.06	3.45×10^{-7}
4-P(ZLL_{60} -co-LA_{20})	60:20	60:20	17500	17500	33400	1.05	5.70×10^{-7}

[a]Theoretical based on stoichiometric feed ratios. [b]Determined by ^{1}H NMR spectroscopy. [c]Determined by SEC. [d]Determined by DOSY NMR spectroscopy.

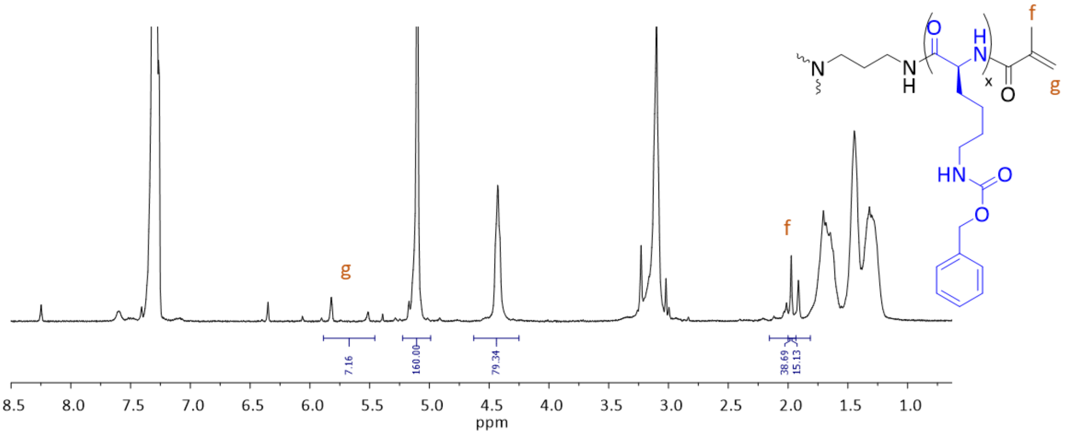

Figure S9. ^{1}H NMR spectrum of 4-P(ZLL_{80})MA (400 MHz, $CDCl_3$/TFA-d).

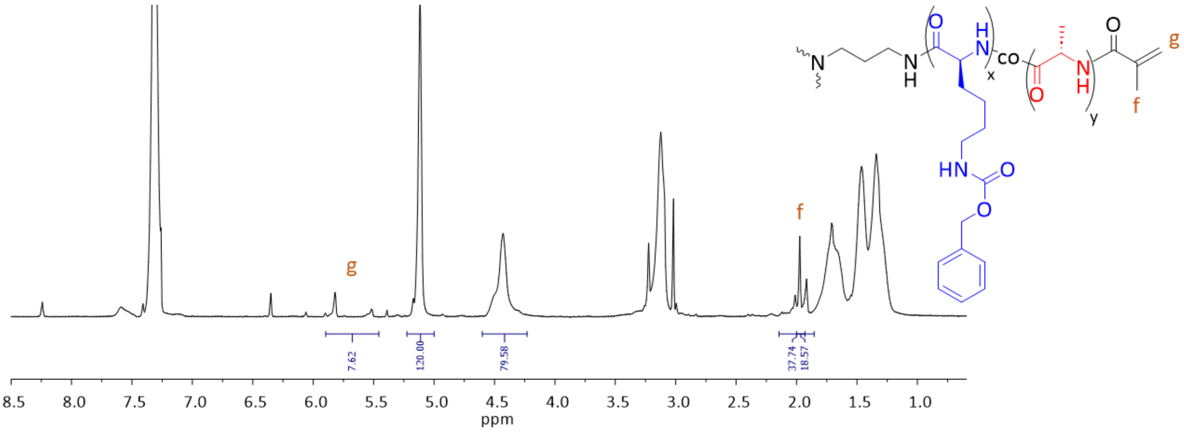

Figure S10. ^{1}H NMR spectrum of 4-P(ZLL_{60}-*co*-LA_{20})MA (400 MHz, $CDCl_3$/TFA-d).

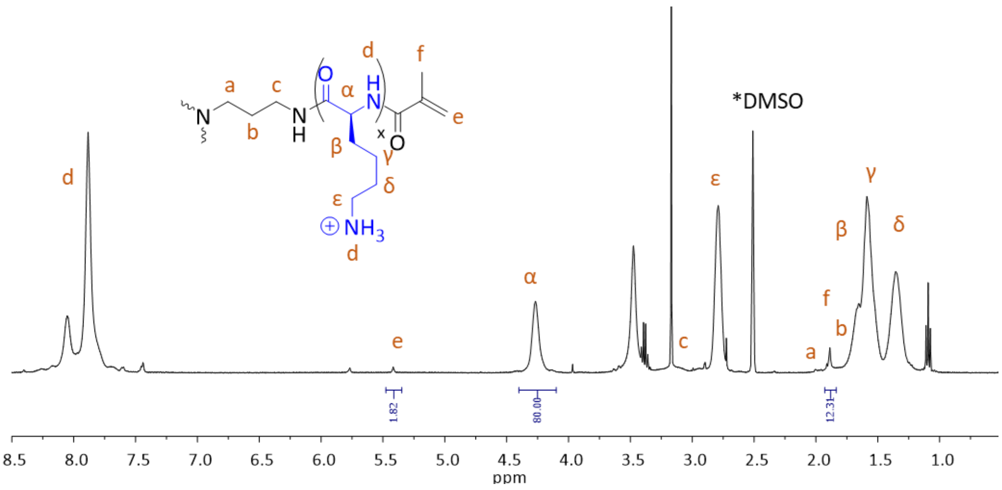

Figure S11. ^{1}H NMR spectrum of 4-P(LL_{80})MA (400 MHz, DMSO-d).

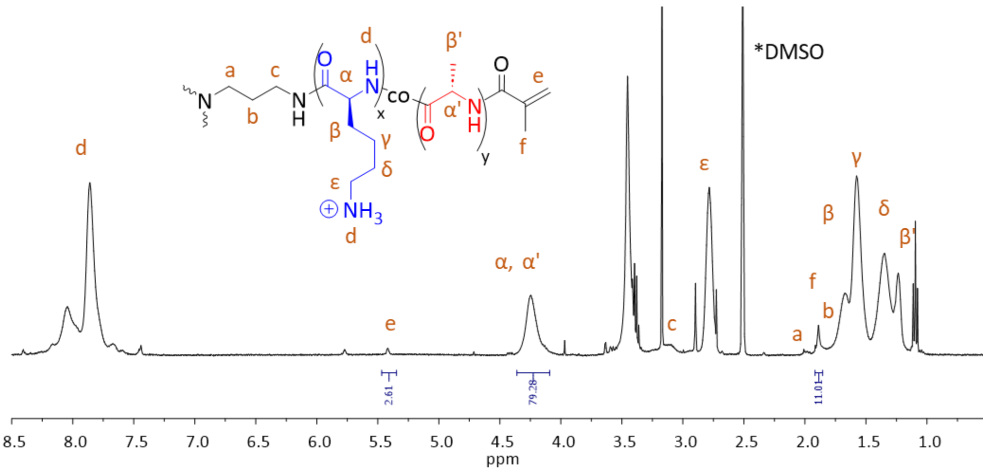

Figure S12. ^{1}H NMR spectrum of 4-P(LL_{60}-*co*-LA_{20})MA (400 MHz, DMSO-d).

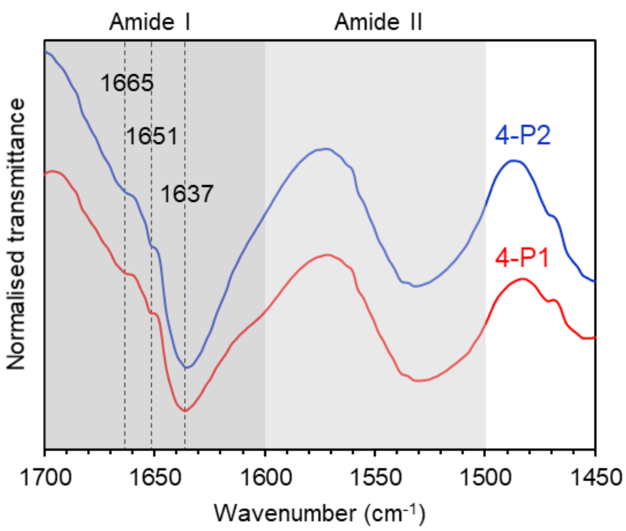

Figure S13. FTIR spectra of 4-P1 and 4-P2 in D_2O.

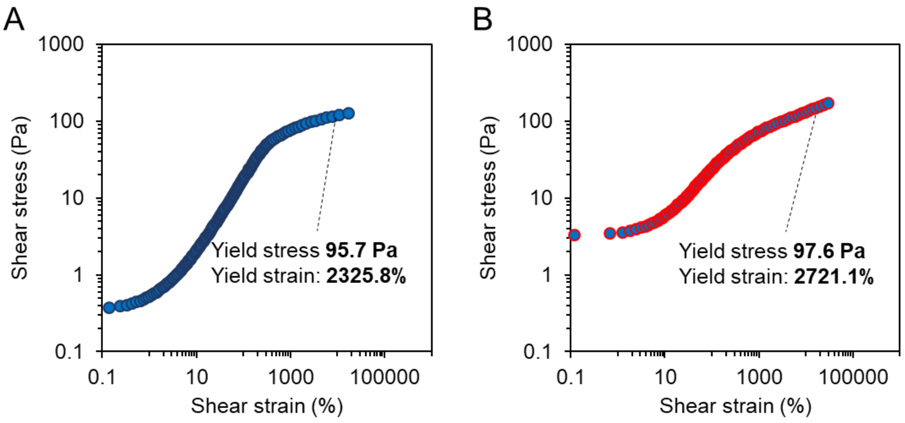

Figure S14. Rheological stress strain sweep displaying yield stress and yield strain of 4-P1 (A) and 4-P2 (B) hydrogels.

Table S2. Hydrogel properties of homopolypeptide and copolypeptide series.

Polymer	M_n[a] (g/mol)	Modulus[b] (Pa)	Equilibrium swelling ratio[c] [Q]
4-P(LL$_{80}$)-MA (4-P1)	19700	5360	19.70±2.80
4-P(LL$_{60}$-*co*-LA$_{20}$)-MA (4-P2)	16300	7520	21.70±3.70

[a]Molecular weight as determined by ^{1}H NMR spectroscopy. [b]Plateaued storage modulus (*G'*) of photocrosslinked hydrogel resin. [c]Equilibrium swelling ratio (Q) calculated from dried and swollen masses of each material.

Table S3. Composition of 4-P1 photoresist.

Reactant	M_n[a] (g/mol)	Amount (mg)	Moles (mmoles)	Mole %	Weight %
4-P(LL$_{80}$)-MA	19700	126.1	0.0064	0.73	37.52
Deionised water	18.02	102	-	-	30.35
N-(2-Hydroxyethyl)acrylamide (HEAAm)	115.13	95.5	0.8295	94.43	28.41
Lithium phenyl-2,4,6-trimethylbenzoylphosphinate (LAP)	294.21	12.5	0.0425	4.84	3.72

Table S4. Composition of 4-P2 photoresist.

Reactant	M_n[a] (g/mol)	Amount (mg)	Moles (mmoles)	Mole %	Weight %
4-P(LL$_{60}$-*co*-LA$_{20}$)-MA	16300	127.6	0.0078	0.89	38.01
Deionised water	18.02	100	-	-	29.79
N-(2-Hydroxyethyl)acrylamide (HEAAm)	115.13	95.3	0.8278	94.16	28.39
Lithium phenyl-2,4,6-trimethylbenzoylphosphinate (LAP)	294.21	12.8	0.0435	4.95	3.81

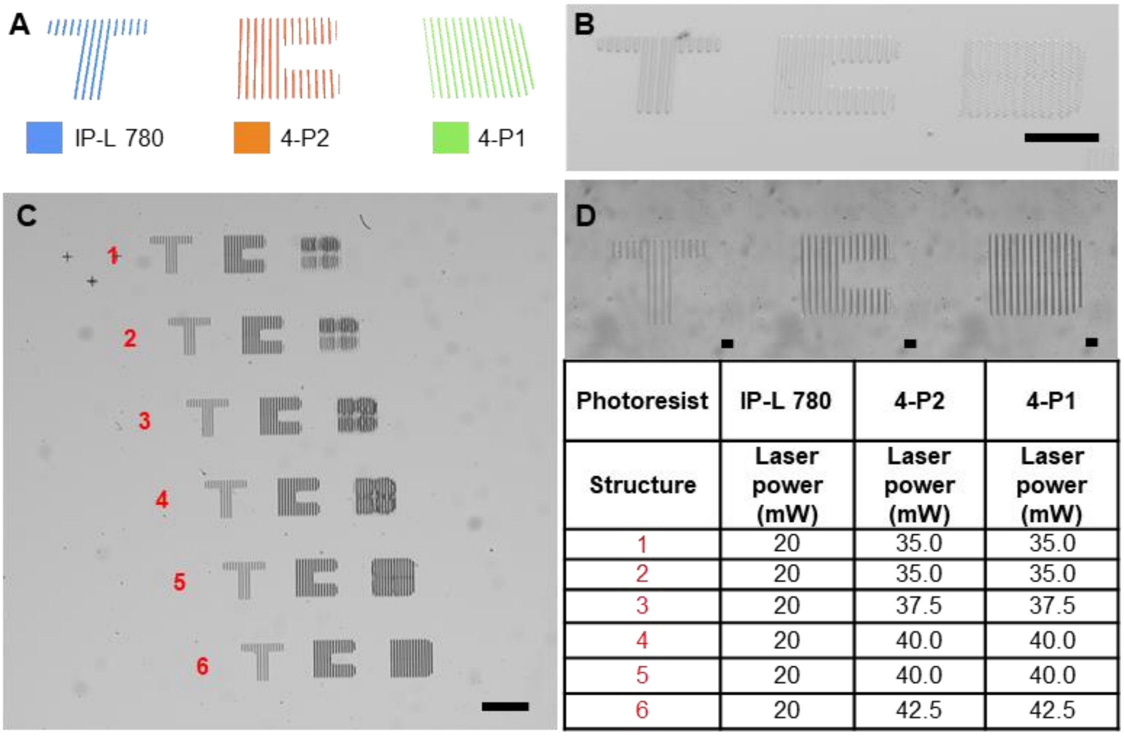

Photoresist	IP-L 780	4-P2	4-P1
Structure	Laser power (mW)	Laser power (mW)	Laser power (mW)
1	20	35.0	35.0
2	20	35.0	35.0
3	20	37.5	37.5
4	20	40.0	40.0
5	20	40.0	40.0
6	20	42.5	42.5

Figure S15. Optimization of fabrication parameters for TCD microstructure. (A) 3D CAD design of TCD letters. (B) SEM image in dry state of structure 6. (C) Optical microscope images of TCD microstructures fabricated with different laser power and scan speed. (D) Higher resolution image of structure 6 (scale bar: (B) 100 μm, (C) 200 μm, (D) 20 μm).

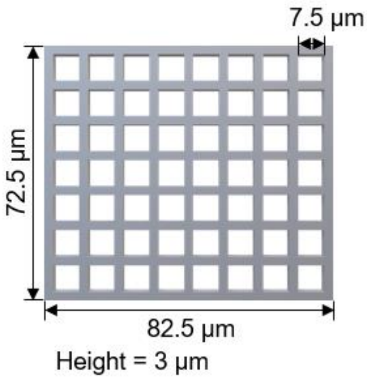

Figure S16. Design of grid used for optimization of printing parameters for both 4P-1 and 4P-2 photoresists.

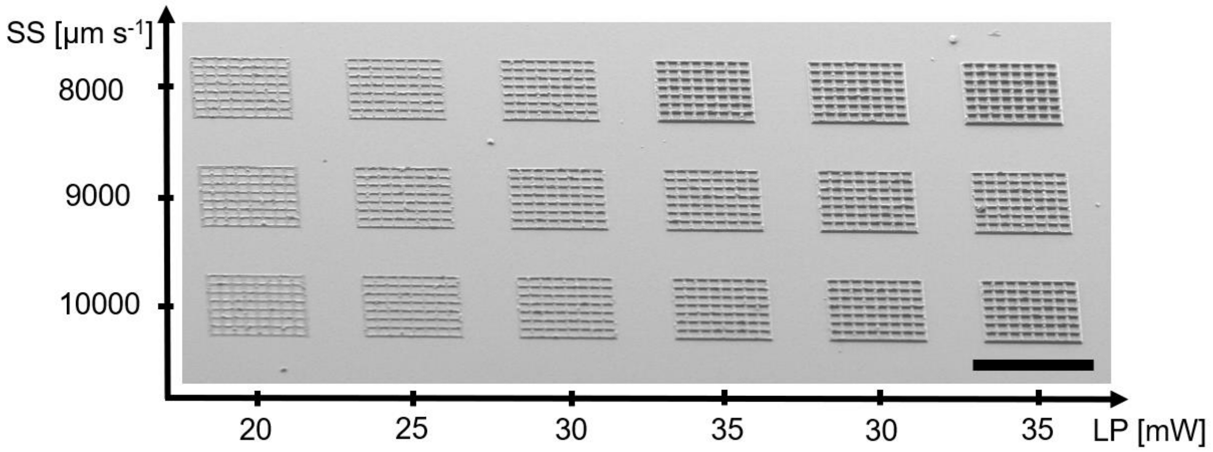

Figure S17. Optimization of laser power (LP) and scanning speed (SS) for 4P-1 microgrids designed with equally spaced 2.5 µm wide lines. SEM was acquired at 45 ° tilt angle. (scale bar: 100 µm).

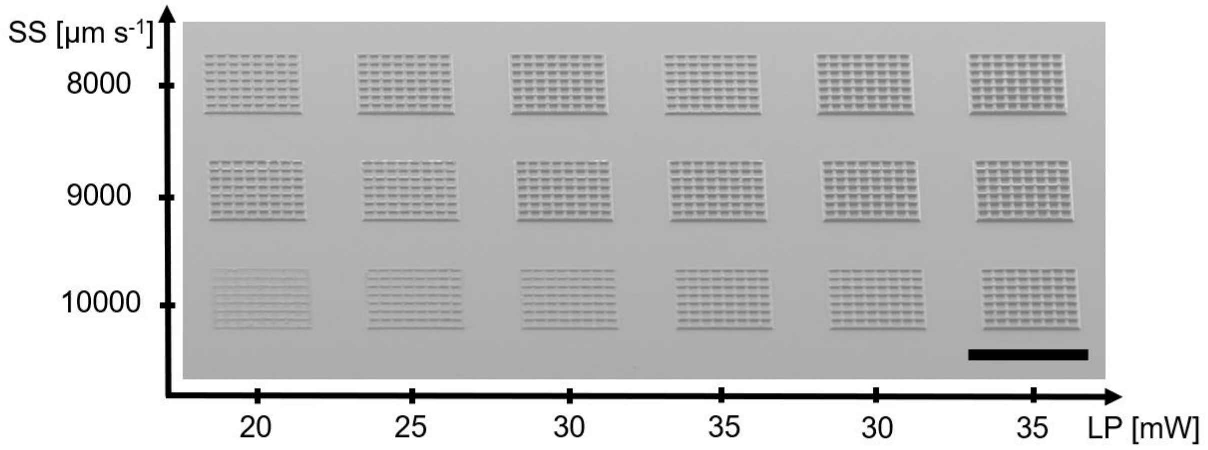

Figure S18. Optimization of laser power (LP) and scanning speed (SS) for 4P-2 microgrids designed of 2.5 µm wide lines equally spaced by 7.5 µm. SEM was acquired at 45 ° tilt angle. (scale bar: 100 µm).

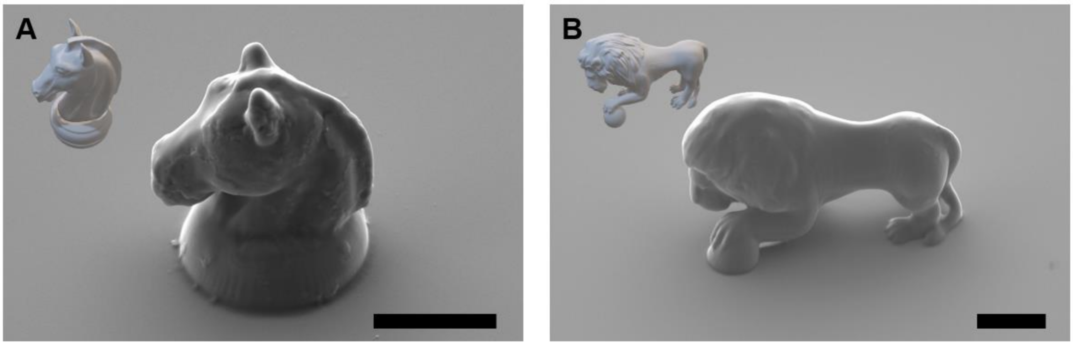

Figure S19. Microstructures fabricated via DLW: (A) SEM of knight chess piece 4P-1 microstructure. (C) SEM of lion statue 4P-2 microstructure. Fabrication parameters: 35 mW laser power and 10000 µm s^{-1} (scale bar: (A) 2 µm, (B) 10 µm).

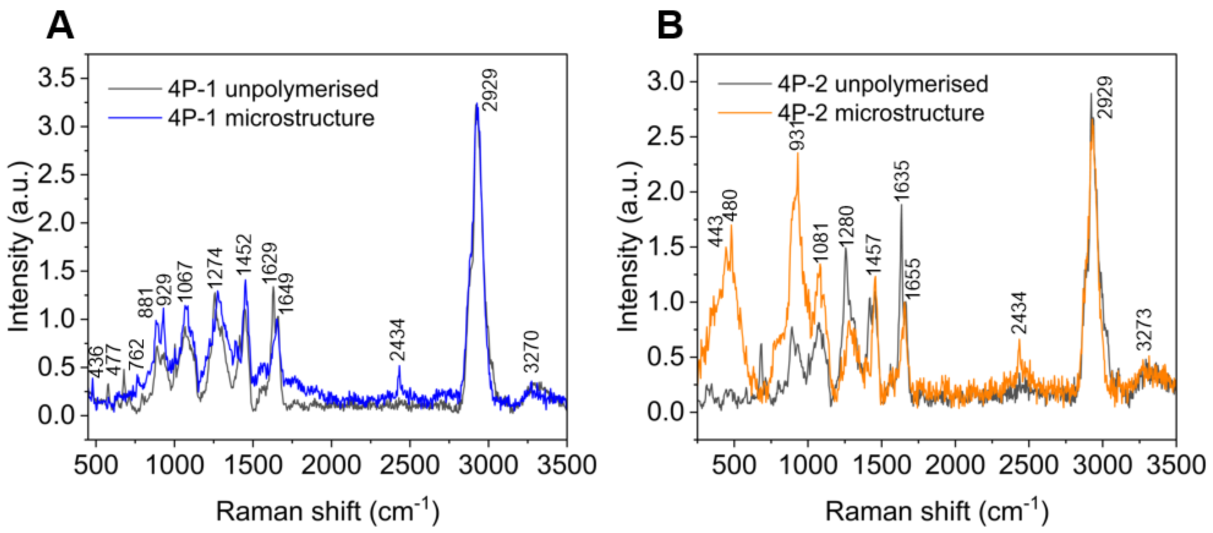

Peak position (cm^{-1})	Assignment
3270	N-H stretch
2929	C-H stretch
1649	C=O stretch (amide I)
1629	C=C stretch
1452	N-H stretch
1274	C-N stretch
1067	C-N stretch
929	C-C-N stretch
477, 436	skeletal deformations

Peak position (cm^{-1})	Assignment
3273	N-H stretch
2929	C-H stretch
1655	C=O stretch
1635	C=C stretch
1457	N-H stretch
1280	C-N stretch
1081	C-N stretch
931	C-C-N stretch
480, 443	skeletal deformations

Figure S20. Raman spectra and assignment of characteristic bands for (A) 4-P1 and (B) 4-P2.

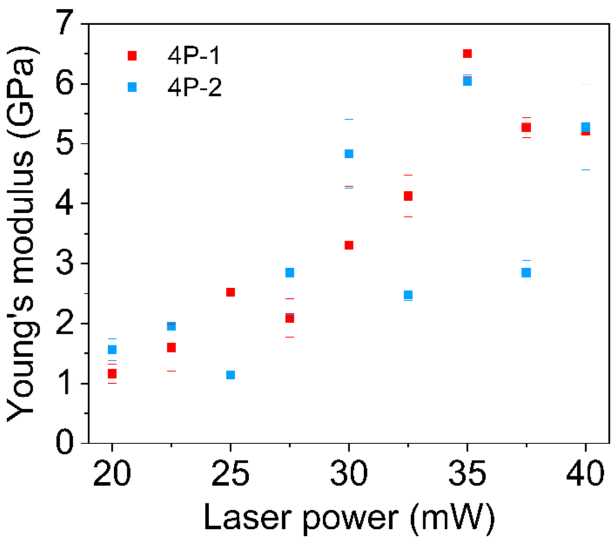

Figure S21. Young’s modulus for 4P-1 and 4P-2 cube arrays in air.

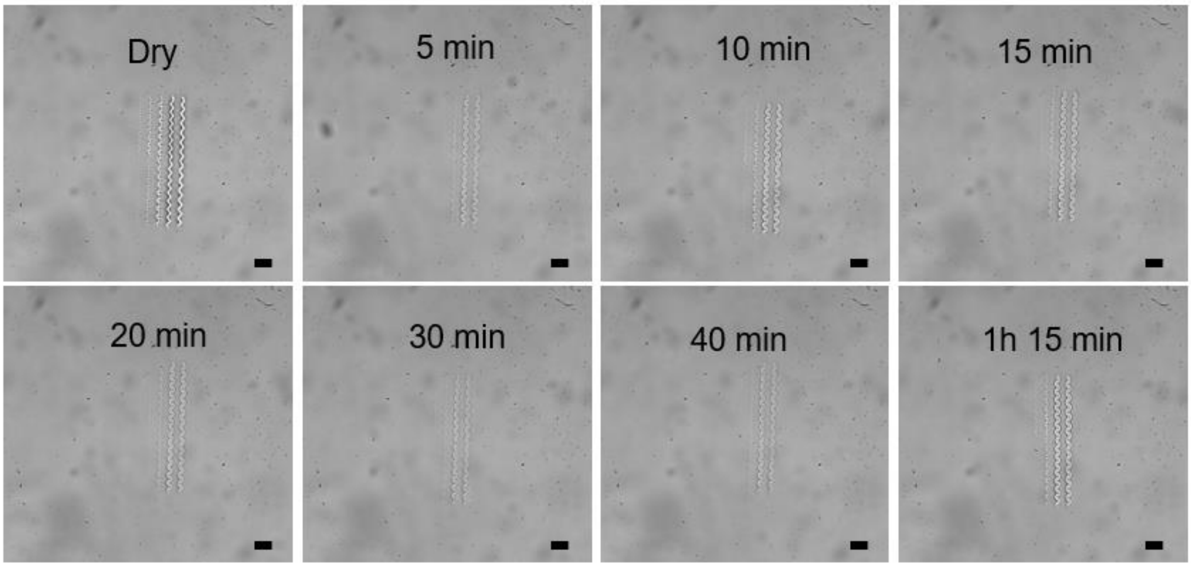

Figure S22. Control degradation of 4-P1 lines in thermolysin solution (0.25 mg/mL) (scale bar: 20 µm). Fabrication parameters: 42.5 mW, 10000 µm s^{-1}.

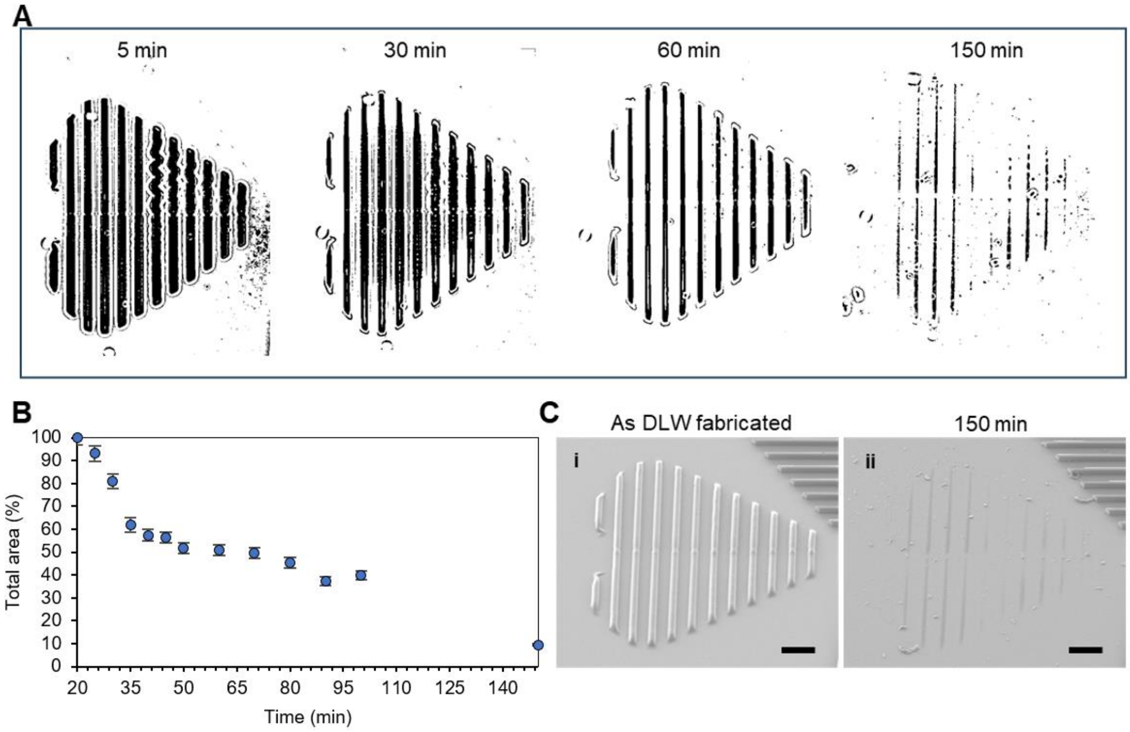

Figure S23. (A) Initial microstructures (5 min) and degradation of 4-P2 microstructures in thermolysin solution (0.25 mg/mL) over time, highlighting degraded area. (B) Graph showing decrease of total area of microstructures (n=3) (scale bar: 50 µm). (D) SEM image of post-degradation (scale bar: 20 µm). Fabrication parameters: 4-P2 (35 mW, 10000 µm s^{-1}).

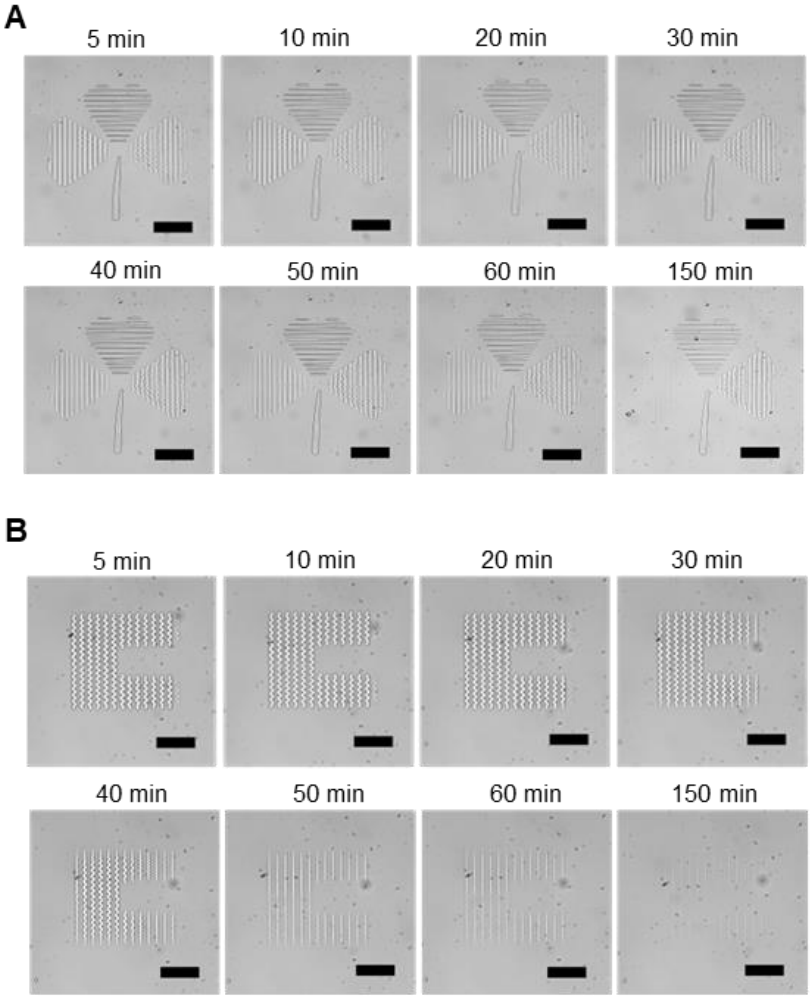

Figure S24. Degradation of clover (A) and C (B) 4-P2 microstructures in thermolysin (0.25 mg/ml) over 150 minutes (scale bar: 100 µm). Fabrication parameters: 4-P1 (35 mW, 10000 µm s^{-1}), 4-P2 (35 mW, 10000 µm s^{-1}), IP-L780 (40 mW, 10000 µm s^{-1}).

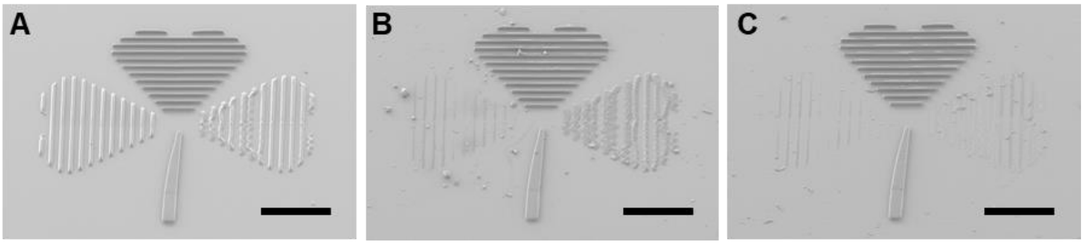

Figure 25. SEM images in dry state of fabricated microstructures (A), after incubation with thermolysin (B), and after incubation with trypsin (C) (scale bar: 100 µm). Fabrication parameters: 4-P1 (35 mW, 10000 µm s^{-1}), 4-P2 (35 mW, 10000 µm s^{-1}), IP-L780 (40 mW, 10000 µm s^{-1}).

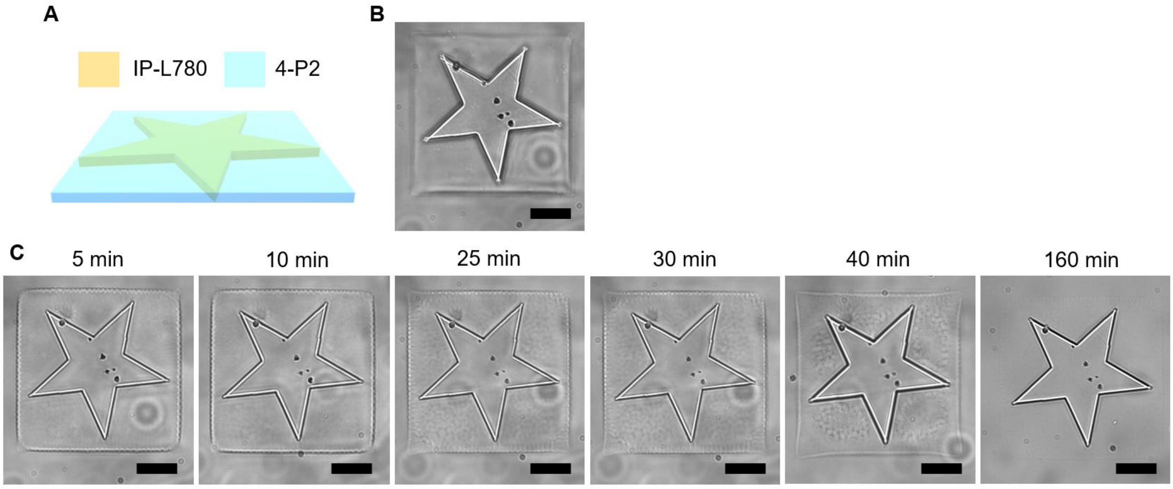

Figure S26. DLW of bimaterial microstructure: (A) 3D CAD design of a non-degradable IPL-780 micro-star encased in an enzyme-degradable cuboid (80 x 80 x 3 µm). (B) In dry state. (C) Degradation of 4-P2 cuboid in thermolysin solution (0.25 mg/mL) over time. Fabrication parameters: 4-P2 (20 mW, 10000 µm s^{-1}), IP-L780 (20 mW, 10000 µm s^{-1}) (scale bar: 20 µm).
